# Suppression of *Wolbachia*-mediated male-killing in the butterfly *Hypolimnas bolina* involves a single genomic region

**DOI:** 10.7717/peerj.7677

**Published:** 2019-10-01

**Authors:** Louise A. Reynolds, Emily A. Hornett, Chris D. Jiggins, Gregory D.D. Hurst

**Affiliations:** 1 Institute of Integrative Biology, University of Liverpool, Liverpool, UK; 2 Department of Zoology, University of Cambridge, Cambridge, UK; 3 Department of Vector Biology, Liverpool School of Tropical Medicine, Liverpool, UK

**Keywords:** Symbiosis, Coevolution, Selfish genetic elements, *Wolbachia*

## Abstract

**Background:**

Sex ratio distorting agents (maternally inherited symbionts and meiotically-driving sex chromosomes) are common in insects. When these agents rise to high frequencies they create strong population sex ratio bias and selection then favours mutations that act to restore the rare sex. Despite this strong selection pressure, the evolution of mutations that suppress sex ratio distorting elements appears to be constrained in many cases, where sex-biased populations persist for many generations. This scenario has been observed in the butterfly *Hypolimnas bolina*, where *Wolbachia-*mediated male killing endured for 800–1,000 generations across multiple populations before the evolution of suppression. Here we test the hypothesis that this evolutionary lag is the result of suppression being a multilocus trait requiring multiple mutations.

**Methods:**

We developed genetic markers, based on conservation of synteny, for each *H. bolina* chromosome and verified coverage using recombinational mapping. We then used a *Wolbachia*-infected mapping family to assess each chromosome for the presence of loci required for male survival, as determined by the presence of markers in all surviving sons.

**Results:**

Informative markers were obtained for each of the 31 chromosomes in *H. bolina*. The only marker that cosegregated with suppression was located on chromosome 25. A genomic region necessary for suppression has previously been located on this chromosome. We therefore conclude that a single genomic region of the *H. bolina* genome is necessary for male-killing suppression.

**Discussion:**

The evolutionary lag observed in our system is not caused by a need for changes at multiple genomic locations. The findings favour hypotheses in which either multiple mutations are required within a single genomic region, or the suppressor mutation is a singularly rare event.

## Introduction

Genetic conflict between selfish genetic elements and their hosts drives evolution, as both host and element evolve to counteract the effects of the other (Hurst & Werren, 2001). These antagonisms are particular strong in the case of selfish genetic elements that distort the sex ratio, such as maternally-inherited symbionts (Hurst & Frost, 2015) and meiotically-driving sex chromosomes (Lindholm et al., 2016). These types of agents can attain high frequencies within a population, giving rise to strongly female-biased population sex ratios (Dyson & Hurst, 2004; Jiggins, Hurst & Majerus, 2000). Such extremely biased sex ratios produce intense Fisherian selection for the production of the rare sex and could potentially drive their host to extinction.

The conflict between sex ratio distorters and their hosts is reflected in the evolution of suppressor genes—genes that impede the sex ratio distorting action of the selfish genetic element and restore sex ratio (Brockhurst et al., 2014). Suppressors of sex chromosome drive (Atlan et al., 1997; Carvalho & Klazcko, 1993; Presgraves, Severance & Wilkinson, 1997) and suppressors of male-killing (Hayashi, Nomura & Kageyama, 2018; Hornett et al., 2006; Majerus & Majerus, 2010; Sasaki, Kubo & Ishikawa, 2002) are known. However, the evolution of suppression is not universal. There are a number of cases where sex ratio distorting elements remain unsuppressed for long periods of time (e.g., X chromosome meiotic drive in *D. pseudoobscura*; male-killing in *Acraea encedon* and *D. innubila*) (Jaenike & Dyer, 2008; Jiggins, Hurst & Majerus, 2000; Policansky & Dempsey, 1978). In *A. encedon*, high prevalence of all female broods and population sex ratios of 20 females per male were first recorded in the 1920s and replicated in subsequent surveys conducted in 1970 and 2000 (Jiggins, Hurst & Majerus, 2000). The persistence of extreme sex ratios that generate very strong selective pressures contrasts with the rapid evolutionary response seen to other strong selection pressures that require *de novo* mutation. Resistance to DDT was observed within 10 years of first use (Sternburg, Kearns & Bruce, 1950), pyrethroids within 2–10 years (Scott, 2017; Vulule et al., 1994), and Bt toxin within 20 years (Tabashnik, 1994). In each case, the response was rapid despite selection being less intense than for suppression of sex ratio distorting agents.

We do not currently understand the factors that constrain the evolution of suppression. A case study that is useful in this regard is the *Hypolimnas bolina*/male-killing *Wolbachia* interaction. In Independent Samoa nearly all females were infected with the male-killer in 2001, producing a 100:1 female-biased population sex ratio that had existed for at least 100 years (Dyson & Hurst, 2004). In Borneo, over 90% of females were infected with the male-killer for more than 80 years prior to the spread of suppression in the 1970s (Hornett et al., 2009). Furthermore, extreme sex ratios have persisted in Tahiti from 1930 to the present day without the evolution of suppression (Hornett et al., 2009). With 10 butterfly generations per year, these data represent cases of at least 800–1,000 generations without evolutionary response. The system is also one where suppression has been observed to spread rapidly when it arises, with migration of an individual carrying the suppressor into the Independent Samoa population leading to a return to a 1:1 Fisherian sex ratio between 2004 and 2006 (Charlat et al., 2007; Hornett et al., 2014).

The contrast of stasis followed by rapid spread of suppression in *H. bolina* provides the opportunity to understand the underlying causes of the constraint. Why was the evolution of suppression slow to evolve despite intense selection pressure favouring male rescue? One possibility is that suppressor mutations are inherently costly as they modify key components of developmental machinery, such as sex determination loci. This would constrain evolution, as there would only be a small subset of mutations capable of both rescuing males and avoiding costs that impede the spread of the suppressor. Hornett, Engelstadter & Hurst (2010) modelled suppressor spread in *H. bolina*, and concluded cost of suppression represents a major barrier to suppressor spread when the male-killer is rare, because rescued males are *Wolbachia* infected and display the CI phenotype, which reduces their value in a population where most females are uninfected (Hornett et al., 2008). However, this barrier is absent at very high male-killer prevalence, as most females are infected and the intensity of Fisherian selection for male-rescue is extreme. Very costly rescue mutations are predicted to spread easily in *H. bolina* populations in which over 90% of females carry the male-killer (Hornett, Engelstadter & Hurst, 2010). Where prevalence is this extreme, mutations can kill females yet spread initially as the males rescued have very high fertility, but they do not then fix as the benefits of male rescue are negatively frequency dependent (Hornett et al., 2014).

Costs of suppression thus represent poor explanations for the evolutionary lag in this system. This view leads to two further hypotheses with respect to constraint. The first is that a single locus controls suppression but the suppressor mutation itself occurs rarely and involves significant changes in e.g., gene structure or function. The second is that the suppressor phenotype involves mutations at multiple loci that only permit male survival when acting in concert. The former hypothesis is rejected where multiple genomic regions are required for male survival, whereas the identification of a single genomic region is consistent with, though not proof of, a rare form of mutation being required to allow male survival.

We know that suppression in *H. bolina* is zygotically-acting and dominant (Hornett et al., 2006). Previous studies have shown that a region of chromosome 25 is necessary for male survival in the presence of a male-killing *Wolbachia* strain (*w*Bol1), with all 60 surviving sons inheriting a 10 cM region of chromosome 25 (Hornett et al., 2014). This genomic region also showed evidence of intense recent selection across the time of suppressor spread. However, we do not know whether any further loci exist that are also necessary for male survival. In this paper, we develop markers for each of the *H. bolina* chromosomes and use segregation analysis to determine whether the genomic region identified on chromosome 25 is sufficient for suppression, or whether other loci within the *H. bolina* genome also enable male-killing suppression.

## Materials & Methods

There are 31 chromosomes in the *H. bolina* genome (Robinson, 1971). A locus necessary for suppression has been identified on chromosome 25 and a further 7 autosomes have been excluded from possessing loci necessary for suppression (Hornett et al., 2014). To assess whether any additional loci within the *H. bolina* genome are required for male-killing suppression, markers were designed to cover all 31 chromosomes. Marker segregation was tracked in mapping families to determine whether a particular linkage group contained a locus necessary for male survival.

### Marker development

The high levels of gene order synteny found amongst the Lepidoptera (Pringle et al., 2007) allows markers to be developed to cover all 31 chromosomes based on their location and non-paralogous nature in *Biston betularia*, *Melitaea cinxia*—species with 31 chromosome pairs and for which linkage maps are available, and also *Bombyx mori* with 28 chromosomes pairs (Ahola et al., 2014; Duan et al., 2010; Van’t Hof et al., 2013). The coding sequence of identified genes acquired from the *B. mori* database (SilkDB) was blasted against a local database to provide *H. bolina*-specific sequence. To create the local database, DNA extracted from a Thai female butterfly was used to create two Illumina paired end libraries (TruSeq fragment library and Nextera large-insert library), which were sequenced on the Illumina HiSeq platform. Reads were assembled using CLC Genomics Server to create a draft *de novo* assembly, which was used to create a custom, local nr BLAST database in Geneious Pro v. 5.6.6. Retrieved *H. bolina* sequence was aligned to *B. mori* coding sequence to infer intron/exon boundaries, so that primers could be designed to amplify intronic regions to develop informative markers, which were then sequenced through the Sanger method. Marker information is detailed in Table S1.

### Association of autosomes with suppression

The *Wolbachia*-infected mapping families described in Hornett et al. (2014) were used to assign each marker to a linkage group (segregation pattern in daughters) and to assess each marker for association with suppression (segregation pattern in sons). The families were created using a reciprocal backcross design whereby a *Wolbachia*-infected female, homozygous for the suppressor, was crossed to a male lacking the suppressor. The female originated from the Philippines, where the suppressor is ubiquitous, whilst the male was derived from Moorea, French Polynesia where the suppressor is not present. This cross produced *F*_1_ offspring that were *Wolbachia* positive and heterozygous for the suppressor.

An *F*_1_ female was then backcrossed to an uninfected Moorean male lacking the suppressor to create the female-informative mapping family. As there is no recombination in female Lepidoptera (Turner & Sheppard, 1975), this ‘female-informative’ mapping family can be used to determine whether a particular linkage group is necessary for suppression: a marker would show Mendelian segregation in *F*_2_ female offspring but be present in all *F*_2_ male offspring. To validate that the selected markers covered all 31 *H. bolina* chromosomes, the segregation pattern of the *F*_2_offspring in females was assessed to ensure that all 30 autosomal markers had a unique pattern of segregation using the package rQTL (Broman et al., 2003).

To determine whether a linkage group contained a locus necessary for male survival, marker sequence was first obtained in the *F*_1_ parents of the female-informative family. Markers that contained SNPs that were heterozygous in the female parent (heterozygous for the suppressor/non-suppressor background) but homozygous in the male parent (no suppression) were then sequenced in the *F*_2_ offspring (*n* = 20 daughters, *n* = 7 sons). Markers that were present in all sons but segregated in female offspring were considered to be associated with suppression (Table S2 and S3). Grandparents were also sequenced to establish which of the segregating alleles was derived from the Filipino (suppressor-carrying) population and which was derived from the Moorean (suppressor absent) population.

### Is a secondary suppressor locus located on the Z chromosome?

The reciprocal male-informative family was created by crossing an *F*_1_ male (*Wolbachia*-infected, heterozygous for the suppressor) with a Moorean *Wolbachia*-infected female lacking the suppressor. This family was used for mapping Z-linked traits. Segregation of the Kettin gene, a well-established Z chromosome marker in Lepidoptera, was tested in this male-informative family (paternal parent is homozygous for the Z chromosome). A SNP marker that was heterozygous in the paternal parent and hemizygous in the maternal parent was sequenced in sons (*n* = 16). Cosegregation for this marker with male survival was assessed. Involvement of the Kettin genomic region would be implicated if the paternal type is overrepresented in surviving male offspring.

## Results

### Do markers represent all 30 autosomes?

**Figure 1:**
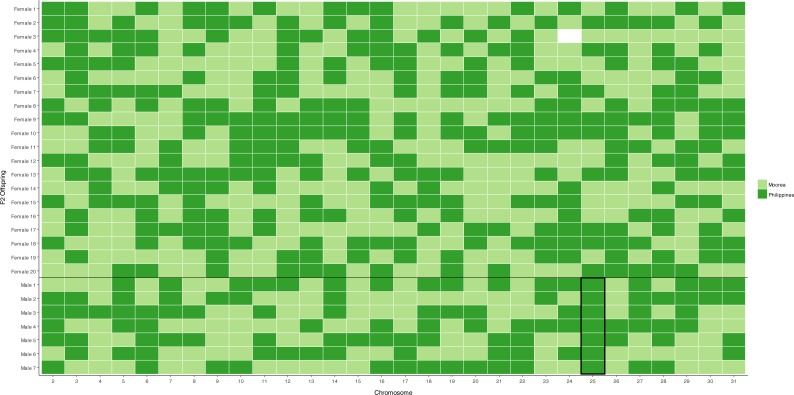
Segregation of autosomal markers into 20 daughters (above) and 7 sons (below) in the female informative family. Dark green indicates inheritance of the Filipino (suppressor population) allele, Light green inheritance of the Moorea (non-suppressor) allele from the mother, missing data left blank. The black box around the chromosome 25 males highlights segregation of the Filipino-derived (suppressor population) allele into all surviving male offspring.

The 30 candidate autosomal markers were sequenced in the grandparents and parents in the female-informative family, haplotype variants in the parents were then identified, along with their population source (Table S2). The segregation pattern of these markers into *F*_2_ female progeny was assessed to test that each marker represents a unique linkage group as predicted by synteny. Each of the 30 markers showed segregation patterns consistent with Mendelian inheritance into daughters, and a unique segregation pattern was shown for each marker in the female offspring of the female-informative family. Independent segregation patterns of the 30 markers was verified with the package rQTL (Fig. 1, Table S3).

### Is a secondary suppressor locus located on an autosome?

A chromosome necessary for male survival is evidenced by a single allele of Filipino origin (where the suppressor is present) segregating into all surviving males tested. This was only observed for the chromosome 25 marker, which has previously been identified as containing a locus necessary for the suppression of *Wolbachia*-induced male-killing (Hornett et al., 2014). For the 29 other markers, the chromosome derived from the Filipino (suppressor) population was never present in more than five of the seven male offspring, implying that loci on these chromosomes are not required for male survival (Fig. 1, Table S3).

### Is a secondary suppressor locus located on the Z chromosome?

A marker on the Z-linked Kettin gene was found to segregate randomly in sons of the male-informative family, with 8 sons carrying the Filipino-derived copy of the marker, and 8 carrying the Moorean-derived copy (Table S2).

## Discussion

The *H. bolina*/male-killing *Wolbachia* interaction represents a case in which there has been considerable evolutionary lag before suppression of male-killing evolved in the butterfly host (Charlat et al., 2007; Dyson & Hurst, 2004; Hornett et al., 2009). The combination of very strong selection for restoration of the sex ratio, and a 800–1,000 generation lag period observed before it occurred, indicates that the evolution of suppression is subject to constraint. This case reflects evolutionary lag observed in other sex ratio distorter/host interactions, but uniquely the spread of suppression that occurred permits investigation of the causes of constraint. In this paper, we tested the hypothesis that the evolutionary lag was associated with a requirement for mutations at two or more different genomic locations for male rescue.

A genomic region on chromosome 25 was previously identified as necessary for male survival in the presence of *Wolbachia w*Bol1 (Hornett et al., 2014). In this paper, we evaluated whether this region is by itself sufficient to suppress male-killing or whether secondary loci exist elsewhere in the *H. bolina* genome that are also necessary for male survival. We were able to exclude the existence of secondary suppressor loci on all the other *H. bolina* autosomes and found no evidence for the existence of a suppressor locus on the Z chromosome. These data reveal that the evolution of suppression of male-killing in *H. bolina* is the result of changes in the single genomic region previously identified on chromosome 25.

Why has suppression taken so long to evolve? The evolutionary lag occurs in the context of high levels of standing genetic variation in the species (for introns, *π* = 0.5–1.2% in isolated island populations (Hornett et al., 2014)). Thus, the suppressor trait represents *de novo* mutation from an already variable gene pool. The broad geographic range and large size of SE Asian *H. bolina* populations (Clarke & Sheppard, 1975) suggests that the mutational event/s that generate suppression are very rare events. There are two hypotheses to explain this rarity. In one model, the evolution of suppression is contingent on changes at multiple loci within the chromosome 25 region identified as necessary for suppression. In the other, there is a single event but it is a complex form of rare mutation.

In this system, a single genomic region is required for male rescue. Single locus suppression is also inferred through segregation patterns in other male-killer host interactions (Majerus & Majerus, 2010), and is also widely found in suppression of sex chromosome meiotic drive (Tao et al., 2007). Sex determining systems represent a target for male-killers (Sugimoto et al., 2012), and the core nature of these pathways may be the source of constraint. Previous work noted that the key sex determining locus *doublesex* was located in the chromosome 25 region in other Lepidoptera (Hornett et al., 2014), and this locus is generally important across insect sex determination. Future efforts to understand the constraint will focus on determining whether *doublesex* evolution underpins suppression in this system, identifying the genetic nature of the change that produces suppression, and explaining the constraint we have observed.

## Conclusions

One hypothesis for the prolonged lag between presence of high prevalence male-killing and the evolution of suppression is that the suppression phenotype requires mutations at more than one genetic locus. Contrary to this hypothesis, we found no evidence that any other regions of the *H. bolina* genome, other than a previously-identified region on chromosome 25, are necessary for male-killing suppression to take place. We therefore conclude that suppression of *Wolbachia*-mediated male-killing in *H. bolina* involved changes in a single genomic region, located on chromosome 25. Future research will be required to determine if this is because of a single complex change or multiple changes within this region.

##  Supplemental Information

10.7717/peerj.7677/supp-1 Table S1 Description of *H. bolina* markers used in this study, including conditions for PCR and accession numbers

10.7717/peerj.7677/supp-2 Table S2 Sequence accession numbers for grandparents and parents in the mapping families

10.7717/peerj.7677/supp-3 Table S3 Segregation pattern of marker variants in the female informative family

10.7717/peerj.7677/supp-4 Dataset S1 Fasta file of marker sequence data for mapping parents and grandparents

## Additional Information and Declarations

### DNA Deposition

The following information was supplied regarding the deposition of DNA sequences: The marker sequences and variants are available at Genbank: MK481229.1–MK481251.

### Data Availability

The following information was supplied regarding data availability: Marker data, accession numbers and SNP segregation patterns are available in Tables S1–S3.
